# Design and Application of Mixed Natural Gas Monitoring System Using Artificial Neural Networks

**DOI:** 10.3390/s21020351

**Published:** 2021-01-07

**Authors:** Jinlei Wang, Bing Li, Bingjie Lei, Peiyuan Ma, Sai Lian, Ning Wang, Xin Li, Shaochong Lei

**Affiliations:** Department of Microelectronics, School of Electronics and Information Engineering, Xi’an Jiaotong University, Xi’an 710049, China; wjljdwdz@stu.xjtu.edu.cn (J.W.); libing888@stu.xjtu.edu.cn (B.L.); leibingjie@mail.xjtu.edu.cn (B.L.); mpy1995@stu.xjtu.edu.cn (P.M.); lian_sai@stu.xjtu.edu.cn (S.L.); wangning07@stu.xjtu.edu.cn (N.W.); lx@mail.xjtu.edu.cn (X.L.)

**Keywords:** natural gas, monitoring system, neural network, sensor array

## Abstract

Natural gas component analysis is one of the significant technologies in the exploitation and utilization of natural gas. A stable and accurate online natural gas monitoring system is necessary for the gas extracting industry. We have developed an online monitoring system of natural gas with a novel hardware architecture. It improves the dependability and maintainability of the system. A specific instruction set is designed to facilitate the coordination of software and hardware. To reduce the sample noise, the exponentially weighted moving average (EWMA) method is used to preprocess the real-time raw data of the sensor array. A tailored neural network is designed for calibration. And the relationship between the performance and the structure of the gas neural network is demonstrated to find the optimal solution for accuracy and hardware scale. The design not only focuses on the optimization of individual components but also focuses on system-level improvement. The system has been running stably for several months in the gas fields. It meets the requirements of stability, ease of use, maintainability, and online monitoring in industrial applications.

## 1. Introduction

Natural gas is more energy-efficient and eco-friendly than coal, and its proportion in the various energy sources consumed in human production activities has increased year by year [1,2]. Natural gas is extracted via a series of physical and chemical operations. The extracted natural gas is a mixture of methane, ethane, propane, and other gases [3]. To ensure the heating quality of natural gas and meet the environmental protection requirements, the composition analysis of natural gas is an essential procedure in the exploitation of natural gas [4,5]. Therefore, the study of natural gas monitoring systems is of great significance for natural gas utilization.

At present, the widely used natural gas testing device is based on gas chromatography technology [6,7]. The operator collects the gas from the gas field, saves it in the gas storage tank, and then determines the gas composition and the concentration via the column chromatographic separation technique in the laboratory [8,9,10]. This off-line test takes several hours or even longer period [11]. Also, the core element in such systems is the operators with professional technical skills, which is expensive and impossible to be deployed in large numbers. Currently, cheap electrical sensors are widely used in industrial monitoring. The gas monitoring system based on electrical sensors becomes a promising solution for online automatic test [12,13,14]. However, the electrical sensors can not be directly used for gas composition analysis due to the cross-sensitivity for different gases [15,16]. The rise of artificial neural networks(ANN) provides a possible solution to the above problem. The data can be rectified by ANN to eliminate the effect of cross-sensitivity.

The mixed gas monitoring system which is based on the electrical sensor array, computed via the artificial neural network, and implemented by the embedded device is one of the significant trends in current research. Tanghao Jia et al. designed a mixed natural gas identification device based on the infrared gas sensor array, using a multi-layer perceptron neural network (MLPNN) to correct gas concentration [17]. Yufeng Fan et al. developed an early warning system to identify toxic gases through a recurrent neural network (RNN) [18]. Maria Gabriella Xibilia et al. proposed a gas monitoring system for industrial environments, using a gas sensor array whose outputs are further processed by a deep neural network [19]. Areej Shahid et al. utilized SnO${}_{2}$ sensor array to implement an ANN-based concentration detection equipment for CH${}_{4}$ and CO [20]. Fayçal Benrekia et al. designed an FPGA-based multi-layer perceptron gas classifier, using an array of eight micro-hotplate-based SnO${}_{2}$ thin film gas sensors [21]. A. Mishra et al. presented two independent ANN blocks to classify and quantify the gas respectively, realizing real-time monitoring of the gas [22]. Arif Hussain Soomro et al. designed a gas safety monitoring system used in coal mining, using sensors to sense gas concentration(methane, carbon monoxide, temperature, and humidity), using ZigBee wireless network to communicate, and using ANN to predict gas concentration level and alarm hazards [23].

At present, many researchers are interested in the natural gas component analysis based on ANN. Most of the researches focuses on the improvement of recognition accuracy by optimizing the analysis algorithms. However, there is no complete system designed for practical industrial applications, including hardware circuits and assistant software. Existing natural gas monitoring devices are unable to meet the actual demands of the gas field. And it is mainly reflected in the following aspects:

(1) The global natural gas fields are widely distributed, and the application scenarios of natural gas monitoring systems are very different, which range from icy and dry inland basins to hot and humid offshore gas fields [24]. Therefore, dependability and maintainability are the primary requirements of the natural gas monitoring system. However, there are many studies on individual elements such as sensors and data processing algorithms, and little attention has been paid to system design, especially to complex application scenarios;

(2) Embedded systems have the advantages of strong specificity, good real-time performance, and low power consumption. Therefore, gas testing systems are usually based on embedded systems. Many processing algorithms are implemented based on FPGA to achieve good real-time performance. However, the existing natural gas testing systems have poor coordination between software and hardware. Sensor parameters need to be calibrated regularly due to their manufacturing differences and continuous aging during use [25]. The general way is to re-program and configure the FPGA, but it involves additional operations and requires professional skills;

(3) In the gas monitoring system, the gas concentration is converted into the corresponding analog electrical signal by the sensor; then it is converted into a digital signal through the ADC [17]. There are various noises caused by non-ideal factors in this process. It is hard to get a good result by feeding the collected data into ANN directly. It is necessary to preprocess the data. Currently, there are few studies about the data preprocessing method for the online monitoring system.

Based on the above analysis, this paper proposes to build a stable, practical, and maintainable online monitoring system of mixed natural gas, using tailored artificial neural networks based on FPGA. An isolation hardware architecture is adopted to improve the stability and maintainability of the device. The sensor parameters need to be calibrated regularly due to the aging of the sensor array, so we designed a specific instruction set to simplify the calibration. Besides, ANN is used in the system to eliminate the cross-sensitivity of the sensors. The optimal structure of the gas neural network is explored to achieve a trade-off of the hardware scale and the accuracy. Considering the noises of the samples, the exponentially weighted moving average method is used to preprocess the real-time data of the sensor array. The system not only achieves high precision through system-level optimization but also can be deployed in a harsh environment, which meets the needs of industrial applications. At present, similar research is in the stage of theory and experiment in the laboratory. Our device has been running stably for several months in the gas field and reached the design target. And it is the first practical natural gas recognition system based on infrared sensors and neural network technology.

## 2. System Design for Mixed Natural Gas Monitoring

**Figure 1:**
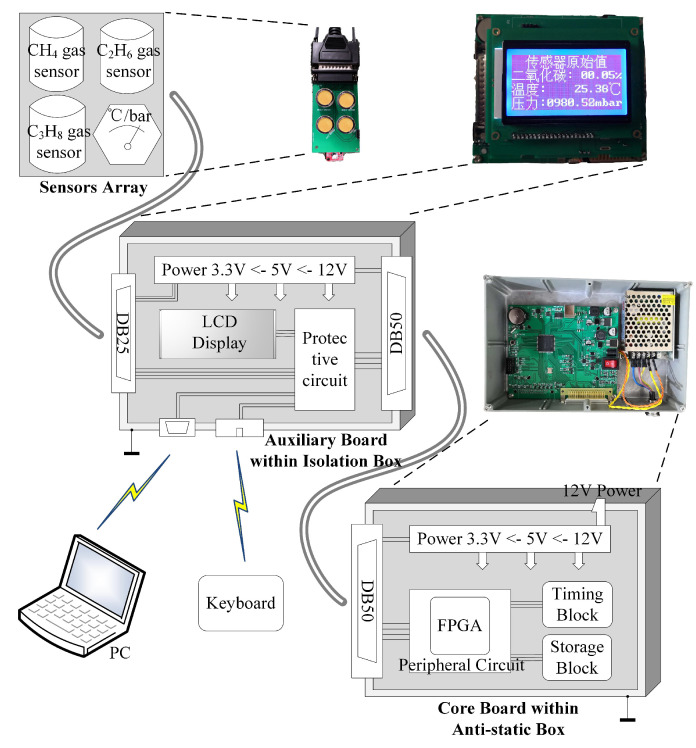
Schematic Diagram of Mixed Natural Gas Monitoring System.

The natural gas monitoring system works in high temperature, low humidity scenarios, so static electricity is more harmful to the hardware circuit in this type of environment. The electrostatic potential generated by the human body is more than 10 kV in the case of low humidity [26]. Also, many other factors lead to electrostatic hazards in the dry environment [26]. The previous gas monitoring device did not have an electrostatic protection design. The device broke down after working in the gas field for half a month. The FPGA is dissected in the laboratory, and it is found that there are signs of electrostatic damage inside the FPGA. Also, the other hardware of the device is not abnormal. The electrostatic protection of the equipment, especially crucial components such as FPGA, is necessary. Therefore, the core circuit of the system is isolated from the peripheral equipment. Figure 1 shows the framework of the mixed natural gas monitoring system. The core circuit (core board) including FPGA is encapsulated in an anti-static box. The electrostatic protection level of the anti-static box has been tested in the laboratory and passed the 6 kV electrostatic discharge immunity test. The auxiliary circuit (auxiliary board) is placed in the isolation box. The sensor array is in the gas chamber and consists of gas sensors. The auxiliary board is the buffer between the core board and the sensor array.

### 2.1. Sensor Array and Isolation Box

According to the composition of the mixed natural gas, the infrared gas sensors produced by Dynament Corporation are selected in this system [27,28]. The sensors have the advantages of fast response speed and slow aging. Besides, a temperature-pressure sensor is added for compensation [29]. The above sensors form a sensor array, and the details are shown in Table 1.

The sensor array is connected to the auxiliary board through the DB25 interface. The auxiliary board consists of the protection circuit, an LCD screen, and various interfaces. A 9-digit numeric keyboard is connected to the auxiliary board. It is convenient for users to manipulate the gas monitoring system. Also, there is a USB2.0 interface on the auxiliary board that interacts with the computer. The historical data can be read and downloaded through this interface. Also, the debugging instructions are sent through it.

The auxiliary board is placed in the isolation box that only provides physical protection. It is the buffer between the core board and the external. On the one hand, the protection circuit ensures that the amplitude of the signals connected to the core board is within a secure range. On the other hand, LCD and various interfaces are used for the interaction between the external and the monitoring system. The cost of its components is low, and the replacement is also convenient.

### 2.2. Core Circuit and Anti-Static Box

There are FPGA (Altera MAX10M50SAE144I7G), power supply module, a storage module, clock management module, etc. on the core board. FPGA performs the ANN calculation and schedules the gas monitoring system to run automatically. The input voltage is converted from 12 V to 5 V and 3.3 V by the power module for the entire monitoring system. The storage module is a 4 Mbits Flash(Adesto Technologies, AT25SF041-SHD-B). The monitoring data and the weight parameters of the neural network are stored in the Flash. DS1302 clock chip is added to reduce the power consumption of the system. It is a low power clock chip of DALLAS, and the power is provided separately by a 220 mAh button battery. The FPGA receives the time information provided by the DS1302, and the entire system is waked up at the setting point in time.

The core board is placed in an anti-static box made of aluminum. The ingress protection grade of the box is IP66. There are only the power interface and the DB50 interface on the anti-static box, and the two interfaces are sealed with silica gel. Therefore, the device has the advantages of good sealing and anti-static. It benefits from the migration of functions such as external interfaces and LCD to a separate auxiliary board, which improves the system stability in a harsh environment.

### 2.3. Cooperation between Hardware and Software

There is a USB2.0 interface on the auxiliary board. And the data path between the PC and the FPGA is constructed through the USB2.0 interface and the DB50 interface. The user can connect the PC with the gas monitoring system. Tailored software is designed to read the historical data, update the neural network parameters, set the system parameters, manually manipulate the equipment, and perform system inspections. Table 2 shows part of the instructions. The instruction length is 32 bits, and it is expressed in hexadecimal. The 31~24 bits of the instruction are function code, the 23~4 bits are data code, the 3~1 bits are reserved bits, and the last bit is the parity bit.

Usually, the monitoring system runs automatically. The system is connected to the computer only when more complicated operations are required. For instance, considering the aging of the sensors, the old neural network parameters are no longer suitable. And the new parameters must be updated to the monitoring system. The aging of the sensors is inevitable, and there is no method to solve the problem. The related parameters need to be calibrated regularly. Therefore, the tailored instruction set in the paper is convenient to update the parameters.

**Figure 2:**
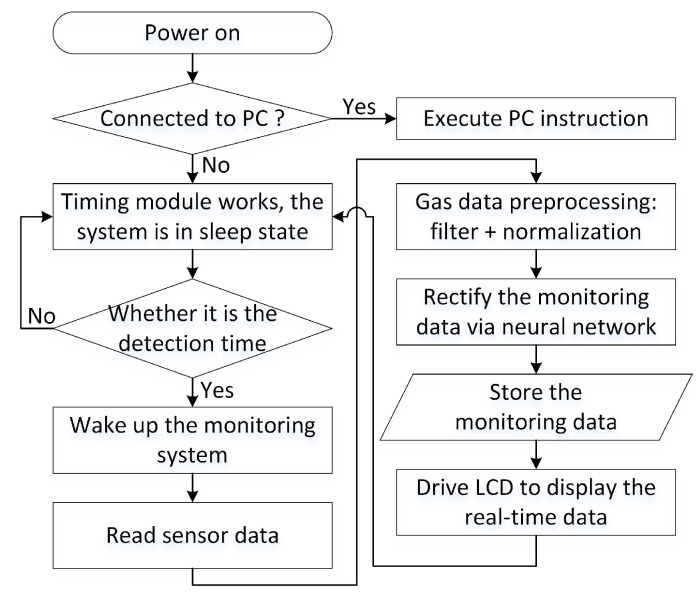
Proposed mixed natural gas monitoring system flowchart.

Figure 2 shows the flowchart of the mixed natural gas monitoring system. When the system is powered on, the device follows the host instructions if it is connected to the host PC. The priority of the computer instructions is the highest. If it is not connected to the host, the system runs automatically in the default mode. To reduce power consumption, only the timing module works all the time, and the entire monitoring system is awakened at the setting time point. The raw data of the sensors is read in, preprocessed, and then rectified by the ANN. The data is saved in the storage module. And the result is displayed by the LCD. Finally, the system enters the sleep state again. Only the timing module works until the start of the next cycle.

The algorithm of the system is implemented on FPGA, and the processing speed is fast because of parallel operation. The hardware design has many advantages, including electrostatic protection, high dependability and stability, low power consumption, good software and hardware cooperation, etc. The system uses wired communication with the host computer, while some advanced measurement and control systems in other fields integrate wireless communication module, which is more convenient. The design needs to be further improved in these aspects.

## 3. Data Processing on FPGA

### 3.1. Data Preprocessing

**Figure 3:**
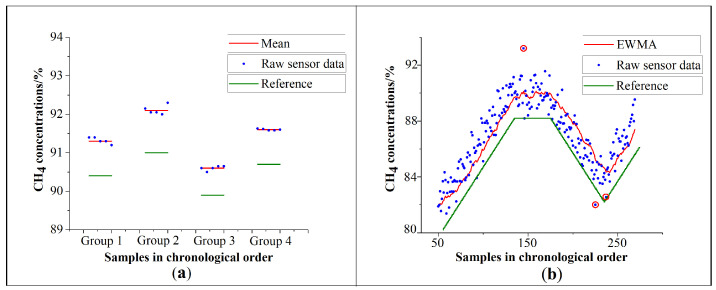
(**a**) Data processed by average method. (**b**) Data processed by EWMA.

When preprocessing the raw data of the gas sensors, there are two scenarios in practical application. One is that the monitoring system performs short-term measurements. Another is that the monitoring system performs real-time, long-term measurement. Therefore, the preprocessing methods should be customized according to specific scenarios. When the application scenario is the short-term measurement, the amount of data is small, and the average data can well reflect the actual value. Figure 3a shows the data of the CH${}_{4}$ sensor in short-term measurement. Four groups of data are measured at four different times. The mean of the raw sensor data can well represent the input methane concentration whose legend is ‘Reference’. By the way, the differences between the mean and the reference value are due to the sensor itself.

Another situation is the real-time, long-term measurement. There are some disadvantages if the average-calculation method is still used. Firstly, it is hard to determine how much data should be averaged. Secondly, it will consume additional storage resources if too much data needs to be saved. Thirdly, the average-calculation method only reflects the recently calculated data and does not incorporate historical data. Therefore, we propose to utilize the exponentially weighted moving average (EWMA) method to preprocess the data. EWMA gives the most recent observation the greatest weight, and all previous observations weights decreasing in geometric progression from the most recent back to the first [30]. This method has been used widely in industrial manufacturing and artificial intelligence. And it requires little computing resource [31]. Let $\theta_{t}$ be the measuring data of the t-th time, β be the weight coefficient that is 0.9 in this design, $x_{t-1}$ be the calculating result of the t − 1th time, and $x_{t}$ be the result of the t-th calculation. The calculation formula of EWMA is given by Equation (1)

(1) $$x_{t}=\beta *x_{t-1}+\left(1-\beta \right)*\theta_{t},t=0,1,2,...$$

The gas concentration changes gradually, so EWMA is suitable for processing this type of data. Figure 3b shows the data of the $CH_{4}$ sensor in long-term measurement. The measurement is continuous. The data processed by EWMA can well reflect the change of the input methane concentration whose legend is ‘Reference’. The sum of squares due to error(SSE) of EWMA is 206.37. Also, the simple moving average(SMA) and the weighted moving average(WMA) are used to process this data as a comparison [32,33]. SSE of raw sensor data, SMA, and WMA are 257.44, 238.65, and 220.11, respectively. The SSE of EWMA is slightly smaller than other methods. Most of all, the gas sensors occasionally sample obviously abnormal data in practice, which are highlighted in red circles in Figure 3b. It is inevitable for gas sensors. EWMA is similar to a filter, which reduces the noise caused by various non-ideal factors and improves the stability of the output of the gas sensors. The preprocessing makes the sensors more suitable for the industrial measurement system that requires stability.

### 3.2. Artificial Neural Network Tailored for Gas Monitoring

**Figure 4:**
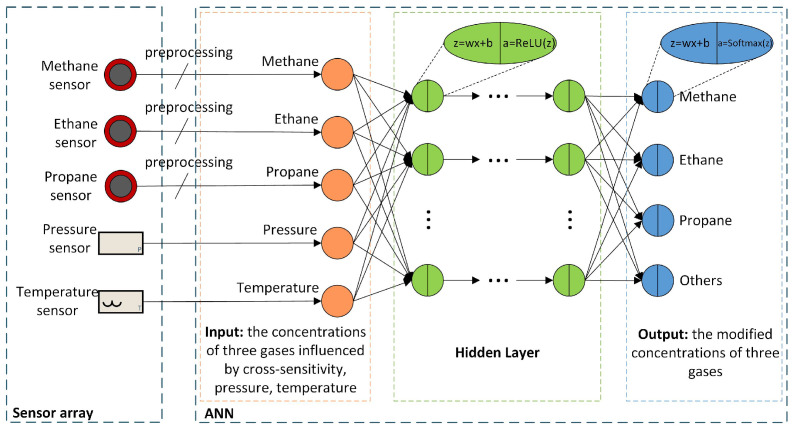
ANN input and the structure of the multilayer perceptron neural network.

In order to eliminate the cross-sensitivity between different sensors and obtain accurate gas concentrations, a multilayer perceptron neural network (MLPNN) is designed for calibration in this work. The advantage of MLPNN is that it does not need to explore the inner relationship between inputs and outputs. Also, the weight parameters can be obtained automatically through the backward propagation (BP) algorithm. The problem can be solved without prior knowledge. Another motivation is that the cross-sensitivity of gas sensors is very complicated. For instance, the methane sensor not only responds to methane but also responds to other alkane gases such as ethane and propane. The impact of multi-component alkane gases on the sensor is hard to distinguish [17]. It is hard to obtain accurate calibration results by prior knowledge or the mathematical model. Therefore, MLPNN becomes a competitive choice. The structure of MLPNN is shown in Figure 4. The input data is raw gas concentrations, temperature, and pressure from the sensor array. And the gas concentrations are preprocessed before feeding into the MLPNN. The activation function of the hidden layer is the rectified linear unit (ReLU) to simplify the hardware implementation. The output layer is the calibrated gas concentrations, and the activation function of the output layer is the softmax function. The softmax function is usually used to the output layer of the neural network of the classification task. Generally, the outputs of softmax are the probabilities of several categories, and the sum of the probabilities is 100%. The function can be applied to the outputs of the gas neural network, but the outputs are no longer probabilities but concentrations. Let $z_{i}$ be the output of the linear calculation of the *i*-th neuron in the output layer, $a_{i}$ is the softmax output of the *i*-th neuron, and the calculation formula of softmax is given by Equation (2):

(2) $$a_{i}=\frac{e^{Z_{i}}}{\Sigma_{k}e^{Z_{k}}}$$

The hyperparameters of a neural network include learning rate, number of iterations, mini-batch size, number of hidden layers, number of hidden layer neurons, etc. Most of the hyperparameters are used to control the training process of the neural network. The training of the neural network is generally on CPU or GPU. And these hyperparameters are useless after training. Therefore, it is necessary to focus on the hyperparameters that could affect hardware implementation. The number of hidden layers and the number of neurons in each hidden layer should be carefully designed. These two hyperparameters determine the scale and performance of the neural network on the FPGA.

**Figure 5:**
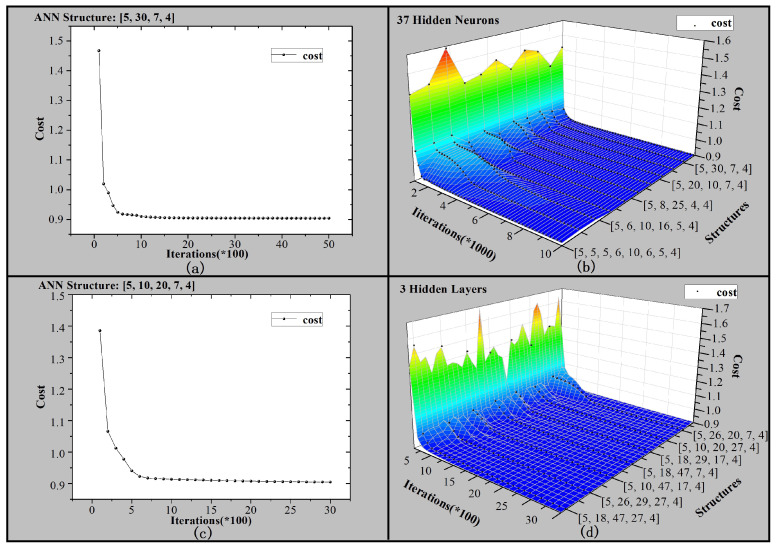
Relationship between costs, iterations and structures of the gas neural networks: (**a**) ANN structure is [5, 30, 7, 4]. (**b**) Different ANN structures with 37 hidden neurons. (**c**) ANN structure is [5, 10, 20, 7, 4]. (**d**) Different ANN structures with 3 hidden layers.

We have measured 4128 groups of data in the laboratory, of which 4000 groups are training set, and the remaining 128 groups are test set. Figure 5 shows the relationship between costs, iterations, and structures. [5, 30, 7, 4] indicates the structure of the neural network. The input layer, two hidden layers, and the output layer consist of five neurons, thirty neurons, seven neurons, and four neurons, respectively. The network structure is denoted in this way in the paper. In order to find a network structure with high accuracy and low hardware scale, various network structures are generated and trained. We use two ways to generate different network structures. Figure 5a is expanded to Figure 5b, keeping 37 hidden neurons unchanged and gradually adjusting the number of hidden layers. Figure 5c is expanded to Figure 5d, keeping three hidden layers unchanged and gradually adjusting the number of neurons. The generation and training of the ANN are performed automatically by computer. So, all kinds of neural networks can be trained in batches.

**Figure 6:**
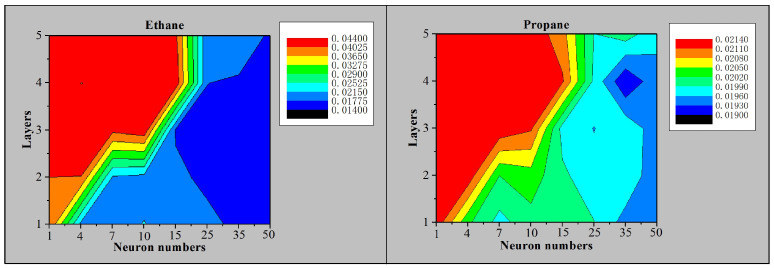
Average error rates of ANN with different layers/neuron numbers.

The trained neural networks are tested on the test set to get the average error rates. Figure 6 shows the average error rates after automatic batch training. Different colors indicate different levels of error rate. As the neuron number increases, the error rate decreases significantly. But the error rate no longer decreases after the neurons increases to a certain number. Besides, the number of hidden layers has little effect on the error rate. The accuracy of methane and other gases has a similar trend. Based on the training results of various networks, the network structure marked as [5, 9, 21, 7, 4] is adopted in our design. Namely, the input layer consists of five neurons; the three hidden layers consist of nine neurons, twenty-one neurons, and seven neurons, respectively; the output layer consists of four neurons. On the one hand, increasing the network scale will not effectively improve accuracy. On the other hand, the resource of hardware implementation is as small as possible. The overall benefit is the highest.

According to the above research, when the gas neural network reaches a certain scale, the structural change has little effect on the accuracy improvement. Therefore, the structure of the gas neural network does not need to be changed even if the detection accuracy decreases due to the aging of the sensors. Only the weight parameters need to be updated regularly to adapt to the aging of the sensors. And the parameters are easy to update through the software instructions designed in this system. It is convenient for the operators in the practical application.

## 4. Field Work of the Mixed Natural Gas Monitoring System and Discussion

The device has been tested both in the laboratory and gas fields. And the monitoring system has been running stably for several months. Due to the cross-sensitivity of gas sensors, the error of the raw data of the sensor is often large in the mixed natural gas composed of alkanes. After the calibration of the neural network, higher recognition accuracy can be achieved.

The experimental equipment in the laboratory includes the gas pipeline system [17] and this monitoring system. The mixed gas is composed of methane, ethane, propane, and nitrogen. Table 3 shows the explanations of the symbols. Table 4 shows the comparison of sensors raw output and the proposed monitoring system output. The concentration of methane is increased from 0% to 100% in 10% steps. The range of ethane and propane is at 0~10%. After the calibration, the maximum errors of the three gases drop to 0.58%, 1.15%, and 1.18%, respectively. The average error of the three gases is 0.24%, 0.33%, and 0.38%, respectively. Therefore, the cross-sensitivity characteristic of the gas sensors is nearly eliminated by the tailored neural network. It proves the feasibility and practicability of the infrared sensor array in natural gas concentration monitoring.

**Figure 7:**
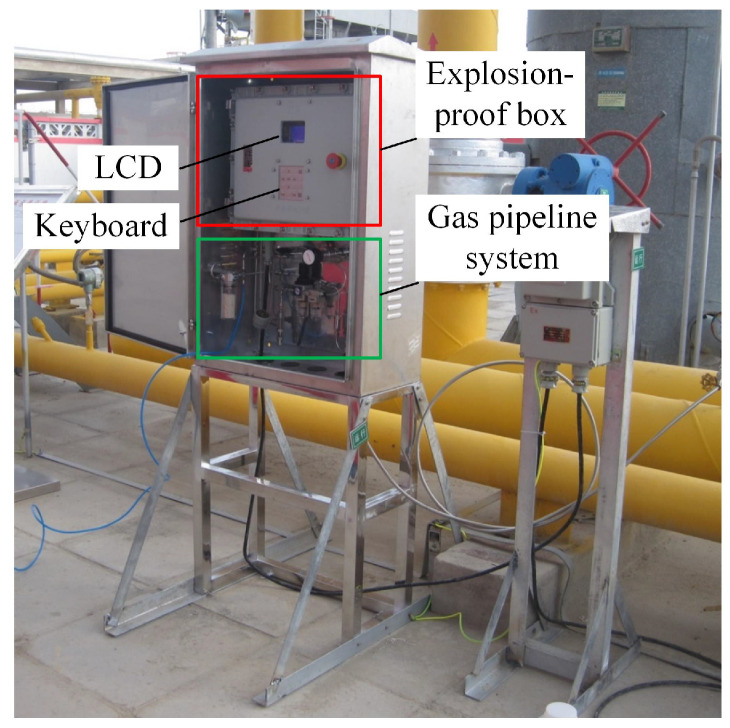
Field work of the mixed natural gas monitoring system.

The monitoring system has been working in Xinjiang Gas Field in China for several months with stable and reliable performance, as shown in Figure 7. The proposed monitoring system is placed in the explosion-proof box for safety. The initial parameters of the neural network are from the training in the laboratory. At the beginning of deployment to the gas field, the network calculation errors are large. The maximum errors of methane, ethane, and propane are 2.1%, 4.33%, and 5.24%, respectively; and the average errors of those are 0.67%, 2.74%, and 2.19%, respectively. It is because the composition of actual natural gas is more complicated. In the process of mining natural gas, the crude gas contains about 15 components. Methane, ethane, propane, and other alkane gases are the main components of natural gas [3]. In the laboratory, the gas composition only involves methane, ethane, propane, and nitrogen. The errors can be essentially regarded as the different sample distributions of the training set and the test set. The neural network is retrained according to the data of the gas field. The new neural network parameters are written into the RAM of the device to improve the recognition accuracy.

There are a few problems with the infrared gas sensors after a long-term operation in the gas field. For instance, we find that some sensors have a zero-drift problem. The sensor data is subsequently processed by ANN, and the weight parameters of ANN are calibrated regularly. Therefore, the measurement accuracy of gas concentration is not affected. And the errors caused by data fluctuation of sensors are eliminated automatically in the process of ANN parameter calibration. It is a good way to calibrate the sensor by calibrating the parameters of ANN, instead of calibrating a single sensor in the conventional method.

The proposed system can realize the online and automatic measurement of natural gas. However, the training of the neural network needs accurate reference samples. And the accurate measurement method of gas composition is gas chromatography. It cannot obtain large-scale samples through gas chromatography in a short time due to the long test cycle and high complexity. Therefore, the update of neural network parameters is a process of gradual adjustment. The improvement of the output accuracy of the system is a process of gradual adjustment. Besides, the neural network parameters need to be calibrated regularly due to the aging and drift of the sensors.

We compare our system with related methods in Table 5. The accuracy of the device is high. And the response time of the infrared electrical sensors is only a few seconds, while gas chromatography and spectrometer need several hours or even days. The sensor data can be processed in real-time due to the parallel computing characteristics of FPGA. And the electrical sensor array requires less volume of natural gas. Most of all, the manufacturing cost and the use cost of the system are lower.

The system is an example of the sensor application. It is of positive significance to promoting the commercial application of such sensors in natural gas monitoring. The infrared sensor is cheaper compared with the gas chromatograph, and the monitoring system based on the infrared sensor array can realize automatic tests. It will have strong market competitiveness. With the wide use of natural gas in the world, the monitoring system based on electrical sensors and neural network technology will receive more attention. How to further improve the accuracy of the test, how to extend the service life of the sensor array, how to eliminate the error caused by sensor aging, how to further improve the automation level of the system will be the directions and focuses of future research.

## 5. Conclusions

Compared with the existing mixed natural gas recognition system, the monitoring system using FPGA-based MLPNN designed in this paper has the following improvements:

(1) We propose a hardware architecture of the natural gas monitoring system that can protect the core circuit well. The extra auxiliary board is a buffer of the core board. Therefore, the developed device can be applied in a harsh environment. And it has the advantage of high stability and low maintenance cost;

(2) The paper proposes to utilize the exponentially weighted moving average method to preprocess the data. EWMA filters out abnormal sampling points and improves the stability of the output of the gas sensors. Also, it consumes little computing resources;

(3) The gas neural network is used to eliminate the cross-sensitivity of the infrared sensors. And the network structure is optimized. It is friendly to hardware implementation. The recognition accuracy of alkane gas reaches 1.5% in the laboratory. Also, a good result is achieved in the field work of the gas field.

The device has been deployed in the gas field and reached the design target. And it is the first practical natural gas analysis system using infrared sensors and neural network technology. There is no relevant product in the market, and the schemes in the literature are still in the stage of theoretical simulation and experiment in the laboratory. The paper proposes a complete set of system architecture, which lays the foundation for the following research.

## Figures and Tables

**Table 1:** Detailed information about the sensors.

Type	Parameter	Value
	Company(Sensor Model)	Dynament(MSHia-P/HCP(HHCP)/NC/5/V/P/XTR/F)
	Resolution	0.1%
Alkane sensor	Detection limit	0–200%
	Selectivity	has cross-sensitivity to alkane
	Response time	30 s
	Company(Sensor Model)	MEAS(MS5803-14BA)
	Pressure Range	0–14 bar
	Pressure Resolution	0.2 mbar
Temperature	Pressure Accuracy	−40–+40 mbar at −40 ${}^{\circ }$C to +85 ${}^{\circ }$C, 0 to 6 bar
-pressure sensor	Temperature Range	−40–+85 ${}^{\circ }$C
	Temperature Resolution	<0.01 ${}^{\circ }$C
	Temperature Accuracy	−0.8–+0.8 ${}^{\circ }$C

**Table 2:** Part of the instruction set.

Instruction	Function
0x8003e011	Set time
0x81002610	Set sampling period
0x82f70001	Set display mode
0x4f000001	Read historical data (raw data)
0x4e000000	Read historical data (rectified data)
0x4d07f010	Update the parameters of neural network
0x20000000	Manually start monitoring
0x21000000	Force to stop monitoring
0xff000000	Soft reset of the system
0xff00ff00	Stop the current task, start receiving host commands
……	……

**Table 3:** Symbol illustrations.

Parameter	Illustration
$C_{in}$	input concentration of each gas
$C_{sout}$	gas sensor output
$S_{1}$	CH${}_{4}$ sensor output
$S_{2}$	C${}_{2}$H${}_{6}$ sensor output
$S_{3}$	C${}_{3}$H${}_{8}$ sensor output
$C_{corr}$	output of the monitoring system

**Table 4:** Comparison of sensor output and the proposed monitoring system output.

$C_{\mathrm{in}}/\%$				$C_{\mathrm{sout}}/\%$			${\mathrm{Errors}\;\mathrm{of}\;C}_{\mathrm{sout}}/\%$			$C_{\mathrm{corr}}/\%$			${\mathrm{Errors}\;\mathrm{of}\;C}_{\mathrm{corr}}/\%$		
${\mathrm{CH}}_{4}$	$C_{2}H_{6}$	$C_{3}H_{8}$	$N_{2}$	$S_{1}$	$S_{2}$	$S_{3}$	$S_{1}$	$S_{2}$	$S_{3}$	${\mathrm{CH}}_{4}$	$C_{2}H_{6}$	$C_{3}H_{8}$	${\mathrm{CH}}_{4}$	$C_{2}H_{6}$	$C_{3}H_{8}$
0	6.1	3.3	90.6	0	12.3	10.1	0	6.2	6.8	0.3	5.96	3.55	0.3	0.14	0.25
9.3	5.3	2.8	82.6	8.3	18.0	16.2	1.0	12.7	13.4	9.42	5.46	2.68	0.12	0.16	0.12
20.6	5.1	6.7	67.6	28.6	32.2	29.9	8	27.1	23.2	20.37	4.73	6.26	0.23	0.37	0.44
30.2	8.6	5.1	56.1	41.1	31.0	28.1	10.9	22.4	23.0	30.78	9.75	6.28	0.58	1.15	1.18
40.4	3.5	5	51.1	51.8	33.4	30.9	11.4	29.9	25.9	40.23	3.2	4.9	0.17	0.3	0.1
50	9.5	4.4	36.1	61.7	78.6	80.1	11.7	69.1	75.7	50.3	9.76	4.07	0.3	0.26	0.33
60.1	9.6	2.1	28.2	71.3	79.8	81.3	11.2	70.2	79.2	60.21	9.87	1.87	0.11	0.27	0.23
69.5	5.2	3.1	22.2	77.4	52.7	52.5	7.9	47.5	49.4	69.4	4.97	3.3	0.1	0.23	0.2
80.2	4.9	4.9	10	83.2	67.1	68.6	3	62.2	63.7	80	5.27	5.58	0.2	0.37	0.68
90	3.4	4.9	1.7	91.5	66.5	66.4	1.5	63.1	61.5	90.14	3.26	4.6	0.14	0.14	0.3
100	0	0	0	100.9	20.9	20.7	0.9	20.9	20.7	99.6	0.19	0.31	0.4	0.19	0.31

**Table 5:** Comparison of our system with other related methods.

Item	Chromatography	Spectrograph	Single Gas Sensor	Our System
Accuracy	high	high	low	high
Online	no	no	yes	yes
Response time	very slow	very slow	fast	fast
Maintainability	low	low	high	high
Require human operation	yes	yes	no	no
Required gas volume	large	large	small	small
Cost	high	very high	very low	low

## Data Availability

Data sharing not applicable.
